# 4,4′-(Cyclo­hexane-1,1-di­yl)dianilinium dichloride monohydrate

**DOI:** 10.1107/S1600536811032995

**Published:** 2011-08-27

**Authors:** Hui-Fen Qian, Wei Huang

**Affiliations:** a College of Sciences, Nanjing University of Technology, Nanjing 210009, People’s Republic of China; b State Key Laboratory of Coordination Chemistry, Nanjing National Laboratory of Microstructures, School of Chemistry and Chemical Engineering, Nanjing University, Nanjing 210093, People’s Republic of China

## Abstract

In the title compound, C_18_H_24_N_2_^2+^·2Cl^−^·H_2_O, both the cation and the water mol­ecule lie on a twofold crystallographic axis. In the cation, the two benzene rings are perpendicular to each other, making a symmetry-constrained dihedral angle of 90°. In the crystal, N—H⋯Cl, O—H⋯Cl and N—H⋯O hydrogen bonds result in the formation of a three-dimensional network.

## Related literature

For related structures, see: Hanton *et al.* (1992 ▶); Qian & Huang (2010 ▶).
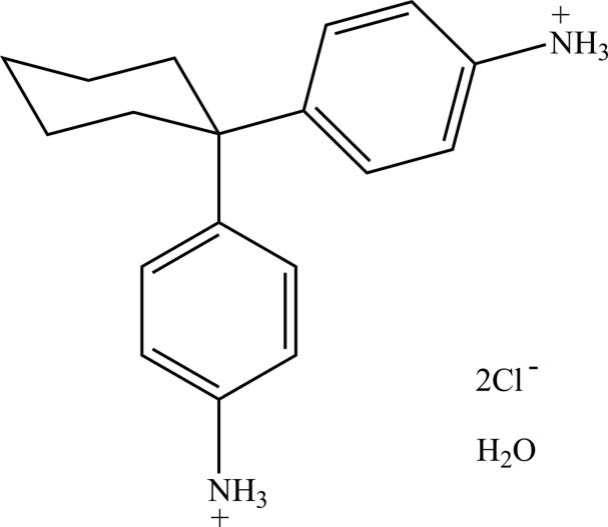


## Experimental

### 

#### Crystal data

C_18_H_24_N_2_^2+^·2Cl^−^·H_2_O
*M* *_r_* = 357.31
Monoclinic, 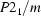

*a* = 8.442 (3) Å
*b* = 9.548 (4) Å
*c* = 12.098 (5) Å
β = 107.085 (5)°
*V* = 932.1 (6) Å^3^
*Z* = 2
Mo *K*α radiation
μ = 0.35 mm^−1^
*T* = 291 K
0.12 × 0.12 × 0.10 mm

#### Data collection

Bruker 1K CCD area-detector diffractometer
Absorption correction: multi-scan (*SADABS*; Bruker, 2000 ▶) *T*_min_ = 0.959, *T*_max_ = 0.965
4705 measured reflections
1735 independent reflections
1158 reflections with *I* > 2σ(*I*)
*R*_int_ = 0.102

#### Refinement

*R*[*F* ^2^ > 2σ(*F* ^2^)] = 0.065
*wR*(*F* ^2^) = 0.212
*S* = 1.10
1735 reflections
126 parameters
H-atom parameters constrained
Δρ_max_ = 0.74 e Å^−3^
Δρ_min_ = −0.51 e Å^−3^

### 

Data collection: *SMART* (Bruker, 2000 ▶); cell refinement: *SAINT* (Bruker, 2000 ▶); data reduction: *SAINT*; program(s) used to solve structure: *SHELXTL* (Sheldrick, 2008 ▶); program(s) used to refine structure: *SHELXTL*; molecular graphics: *SHELXTL*; software used to prepare material for publication: *SHELXTL*.

## Supplementary Material

Crystal structure: contains datablock(s) global, I. DOI: 10.1107/S1600536811032995/ff2022sup1.cif

Structure factors: contains datablock(s) I. DOI: 10.1107/S1600536811032995/ff2022Isup2.hkl

Supplementary material file. DOI: 10.1107/S1600536811032995/ff2022Isup3.cml

Additional supplementary materials:  crystallographic information; 3D view; checkCIF report

## Figures and Tables

**Table 1:** Hydrogen-bond geometry (Å, °)

*D*—H⋯*A*	*D*—H	H⋯*A*	*D*⋯*A*	*D*—H⋯*A*
N2—H2*C*⋯Cl1^i^	0.89	2.70	3.253 (3)	121
N2—H2*C*⋯Cl1^ii^	0.89	2.53	3.252 (3)	139
N2—H2*B*⋯Cl1^iii^	0.89	2.41	3.253 (3)	159
N2—H2*A*⋯Cl1^iv^	0.89	2.46	3.252 (3)	148
O1—H1*E*⋯Cl1^v^	0.85	2.42	3.183 (3)	150
O1—H1*D*⋯Cl1^vi^	0.82	2.38	3.183 (3)	167
N1—H1*C*⋯Cl1^vii^	0.90	2.22	3.101 (3)	165
N1—H1*B*⋯O1^viii^	0.90	1.78	2.678 (6)	172
N1—H1*A*⋯Cl1^ix^	0.90	2.23	3.101 (3)	163

Symmetry codes: (i) 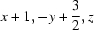; (ii) 
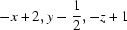
; (iii) 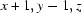; (iv) 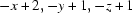; (v) 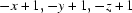; (vi) 
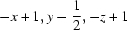
; (vii) 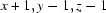; (viii) 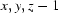; (ix) 
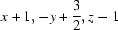
.

**Fig. 1.**
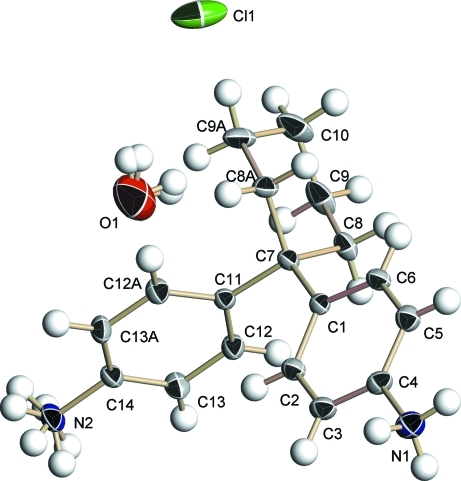
An ORTEP drawing of the title compound with the atom-numbering scheme. Displacement ellipsoids are drawn at the 30% probability level. [Symmetry codes: (A) x, 0.5-y, z]

## supplementary crystallographic information

### Comment

There have been only one related single-crystal structural report on 1,1-bis(4-amino-3,5-dimethylphenyl)cyclohexane (Hanton *et al.*, 1992). We have previously reported the single-crystal structure of a similar compound biphenyl-3,3',4,4'-tetraamine (Qian & Huang, 2010). In this work, we describe the single-crystal structure of hydrochloride salt of 1,1-bis(4-aminophenyl)cyclohexane.

The atom-numbering scheme of the title compound is shown in Fig. 1, while selected bond distances and bond angles are given in Table 1. The two phenyl rings of the title compound are perpendicular to each other with a dihedral angle of 90°. In the crystal packing, N—H···Cl and O—H···Cl hydrogen-bond interactions give rise to a three-dimensional network.

### Experimental

The treatment of 1,1-bis(4-aminophenyl)cyclohexane dissolved in methanol with an excess of hydrochloric acid yields the title compound. Single crystals suitable for X-ray diffraction measurement were obtained after 7 days' slow evaporation of the mother liquid at room temperature in air. Anal. Calcd. For C_18_H_24_N_2_^2+^.2Cl^-^.H_2_O: C, 60.50; H, 7.33; N, 7.84%. Found: C, 60.31; H, 7.55; N, 7.96%.

### Refinement

The non-hydrogen atoms were refined anisotropically, whereas the H atoms bonded with carbon, nitrogen and oxygen atoms were placed in geometrically idealized positions (C—H = 0.93 or 0.97 Å, N—H = 0.89 Å and O—H = 0.82 or 0.85 Å) and refined as riding atoms, with *U*_iso_(H) = 1.2*U*_eq_(C) and 1.2*U*~eq~(N) and *U*ĩso~(H) = 1.5*U*~eq~(O). The hydrogen atoms bonded to atoms O1 and N2 are refined as the model with split positions.

### Crystal data

**Table:**

C_18_H_24_N_2_^2+^·2Cl^−^·H_2_O	*F*(000) = 380
*M**_r_* = 357.31	*D*_x_ = 1.273 Mg m^−^^3^
Monoclinic, *P*2_1_/*m*	Mo *K*α radiation, λ = 0.71073 Å
Hall symbol: -P 2yb	Cell parameters from 1254 reflections
*a* = 8.442 (3) Å	θ = 2.5–24.5°
*b* = 9.548 (4) Å	µ = 0.35 mm^−^^1^
*c* = 12.098 (5) Å	*T* = 291 K
β = 107.085 (5)°	Block, colourless
*V* = 932.1 (6) Å^3^	0.12 × 0.12 × 0.10 mm
*Z* = 2	

### Data collection

**Table:**

Bruker 1K CCD area-detector diffractometer	1735 independent reflections
Radiation source: fine-focus sealed tube	1158 reflections with *I* > 2σ(*I*)
graphite	*R*_int_ = 0.102
φ and ω scans	θ_max_ = 25.0°, θ_min_ = 1.8°
Absorption correction: multi-scan (*SADABS*; Bruker, 2000)	*h* = −8→10
*T*_min_ = 0.959, *T*_max_ = 0.965	*k* = −11→10
4705 measured reflections	*l* = −14→13

### Refinement

**Table:**

Refinement on *F*^2^	Primary atom site location: structure-invariant direct methods
Least-squares matrix: full	Secondary atom site location: difference Fourier map
*R*[*F*^2^ > 2σ(*F*^2^)] = 0.065	Hydrogen site location: inferred from neighbouring sites
*wR*(*F*^2^) = 0.212	H-atom parameters constrained
*S* = 1.10	*w* = 1/[σ^2^(*F*_o_^2^) + (0.1167*P*)^2^] where *P* = (*F*_o_^2^ + 2*F*_c_^2^)/3
1735 reflections	(Δ/σ)_max_ < 0.001
126 parameters	Δρ_max_ = 0.74 e Å^−^^3^
0 restraints	Δρ_min_ = −0.51 e Å^−^^3^

### Special details

**Table:**

Experimental. The structure was solved by direct methods (Bruker, 2000) and successive difference Fourier syntheses.
Geometry. All e.s.d.'s (except the e.s.d. in the dihedral angle between two l.s. planes) are estimated using the full covariance matrix. The cell e.s.d.'s are taken into account individually in the estimation of e.s.d.'s in distances, angles and torsion angles; correlations between e.s.d.'s in cell parameters are only used when they are defined by crystal symmetry. An approximate (isotropic) treatment of cell e.s.d.'s is used for estimating e.s.d.'s involving l.s. planes.
Refinement. Refinement of *F*^2^ against ALL reflections. The weighted *R*-factor *wR* and goodness of fit *S* are based on *F*^2^, conventional *R*-factors *R* are based on *F*, with *F* set to zero for negative *F*^2^. The threshold expression of *F*^2^ > σ(*F*^2^) is used only for calculating *R*-factors(gt) *etc*. and is not relevant to the choice of reflections for refinement. *R*-factors based on *F*^2^ are statistically about twice as large as those based on *F*, and *R*- factors based on ALL data will be even larger.

### Fractional atomic coordinates and isotropic or equivalent isotropic displacement parameters (Å^2^)

**Table:**

	*x*	*y*	*z*	*U*_iso_*/*U*_eq_	Occ. (<1)
C1	0.9232 (4)	0.2500	−0.0868 (3)	0.0294 (9)	
C2	1.0908 (5)	0.2500	−0.0779 (3)	0.0359 (9)	
H2	1.1675	0.2500	−0.0048	0.043*	
C3	1.1484 (5)	0.2500	−0.1747 (4)	0.0417 (10)	
H3	1.2616	0.2500	−0.1659	0.050*	
C4	1.0383 (5)	0.2500	−0.2816 (3)	0.0386 (10)	
C5	0.8702 (5)	0.2500	−0.2956 (3)	0.0425 (10)	
H5	0.7954	0.2500	−0.3694	0.051*	
C6	0.8136 (5)	0.2500	−0.1994 (3)	0.0372 (10)	
H6	0.7000	0.2500	−0.2095	0.045*	
C7	0.8564 (4)	0.2500	0.0183 (3)	0.0319 (9)	
C8	0.7457 (3)	0.1189 (3)	0.0130 (2)	0.0424 (8)	
H8A	0.6611	0.1165	−0.0613	0.051*	
H8B	0.8131	0.0354	0.0190	0.051*	
C9	0.6634 (4)	0.1176 (4)	0.1081 (3)	0.0613 (11)	
H9A	0.7474	0.1105	0.1824	0.074*	
H9B	0.5920	0.0362	0.0991	0.074*	
C10	0.5613 (6)	0.2500	0.1054 (4)	0.077 (2)	
H10A	0.4679	0.2500	0.0358	0.092*	
H10B	0.5181	0.2500	0.1713	0.092*	
C11	1.0029 (4)	0.2500	0.1307 (3)	0.0305 (9)	
C12	1.0744 (3)	0.1265 (3)	0.1817 (2)	0.0403 (8)	
H12	1.0283	0.0417	0.1503	0.048*	
C13	1.2123 (4)	0.1256 (3)	0.2777 (2)	0.0436 (8)	
H13	1.2590	0.0414	0.3101	0.052*	
C14	1.2789 (5)	0.2500	0.3243 (3)	0.0362 (10)	
Cl1	0.35794 (16)	1.02159 (14)	0.60558 (11)	0.1036 (6)	
N1	1.1019 (5)	0.2500	−0.3833 (3)	0.0507 (10)	
H1A	1.1617	0.3283	−0.3818	0.061*	0.50
H1B	1.0163	0.2500	−0.4484	0.061*	
H1C	1.1641	0.1729	−0.3814	0.061*	0.50
N2	1.4267 (4)	0.2500	0.4255 (3)	0.0493 (10)	
H2A	1.5082	0.2037	0.4086	0.059*	0.50
H2B	1.4031	0.2084	0.4846	0.059*	0.50
H2C	1.4584	0.3378	0.4446	0.059*	0.50
O1	0.8669 (6)	0.2500	0.4122 (4)	0.128 (2)	
H1D	0.8043	0.3178	0.3960	0.192*	0.50
H1E	0.7860	0.1950	0.3841	0.192*	0.50

### Atomic displacement parameters (Å^2^)

**Table:**

	*U*^11^	*U*^22^	*U*^33^	*U*^12^	*U*^13^	*U*^23^
C1	0.030 (2)	0.025 (2)	0.032 (2)	0.000	0.0082 (15)	0.000
C2	0.030 (2)	0.039 (2)	0.039 (2)	0.000	0.0105 (16)	0.000
C3	0.035 (2)	0.046 (3)	0.048 (2)	0.000	0.0184 (19)	0.000
C4	0.041 (2)	0.041 (2)	0.041 (2)	0.000	0.0226 (19)	0.000
C5	0.044 (2)	0.046 (3)	0.036 (2)	0.000	0.0099 (18)	0.000
C6	0.028 (2)	0.043 (2)	0.041 (2)	0.000	0.0109 (17)	0.000
C7	0.0252 (19)	0.036 (2)	0.033 (2)	0.000	0.0060 (15)	0.000
C8	0.0352 (16)	0.054 (2)	0.0357 (15)	−0.0139 (14)	0.0078 (12)	0.0014 (13)
C9	0.0405 (18)	0.099 (3)	0.0450 (18)	−0.0282 (19)	0.0127 (14)	0.0056 (18)
C10	0.035 (3)	0.155 (6)	0.045 (3)	0.000	0.018 (2)	0.000
C11	0.0286 (19)	0.035 (2)	0.0291 (19)	0.000	0.0101 (15)	0.000
C12	0.0418 (17)	0.0318 (16)	0.0425 (16)	0.0008 (13)	0.0051 (13)	−0.0019 (12)
C13	0.0439 (17)	0.0334 (17)	0.0456 (17)	0.0044 (13)	0.0007 (13)	0.0073 (13)
C14	0.030 (2)	0.043 (2)	0.033 (2)	0.000	0.0056 (17)	0.000
Cl1	0.1165 (11)	0.1117 (11)	0.1092 (10)	0.0666 (8)	0.0747 (8)	0.0572 (7)
N1	0.053 (2)	0.057 (3)	0.050 (2)	0.000	0.0262 (18)	0.000
N2	0.041 (2)	0.058 (3)	0.039 (2)	0.000	−0.0023 (15)	0.000
O1	0.106 (4)	0.199 (7)	0.067 (3)	0.000	0.007 (3)	0.000

### Geometric parameters (Å, °)

**Table:**

C1—C2	1.388 (5)	C9—H9B	0.9700
C1—C6	1.404 (5)	C10—C9^i^	1.525 (5)
C1—C7	1.535 (5)	C10—H10A	0.9700
C2—C3	1.393 (5)	C10—H10B	0.9700
C2—H2	0.9300	C11—C12^i^	1.384 (3)
C3—C4	1.352 (6)	C11—C12	1.384 (3)
C3—H3	0.9300	C12—C13	1.383 (4)
C4—C5	1.379 (6)	C12—H12	0.9300
C4—N1	1.480 (5)	C13—C14	1.363 (3)
C5—C6	1.382 (5)	C13—H13	0.9300
C5—H5	0.9300	C14—C13^i^	1.363 (3)
C6—H6	0.9300	C14—N2	1.469 (5)
C7—C11	1.546 (5)	N1—H1A	0.8993
C7—C8	1.552 (4)	N1—H1B	0.8990
C7—C8^i^	1.552 (4)	N1—H1C	0.9005
C8—C9	1.508 (4)	N2—H2A	0.8900
C8—H8A	0.9700	N2—H2B	0.8900
C8—H8B	0.9700	N2—H2C	0.8900
C9—C10	1.525 (5)	O1—H1D	0.8217
C9—H9A	0.9700	O1—H1E	0.8491
			
C2—C1—C6	116.2 (3)	C10—C9—H9B	109.4
C2—C1—C7	123.4 (3)	H9A—C9—H9B	108.0
C6—C1—C7	120.4 (3)	C9^i^—C10—C9	112.0 (4)
C1—C2—C3	122.3 (4)	C9^i^—C10—H10A	109.2
C1—C2—H2	118.9	C9—C10—H10A	109.2
C3—C2—H2	118.9	C9^i^—C10—H10B	109.2
C4—C3—C2	119.5 (4)	C9—C10—H10B	109.2
C4—C3—H3	120.3	H10A—C10—H10B	107.9
C2—C3—H3	120.3	C12^i^—C11—C12	116.8 (3)
C3—C4—C5	120.7 (4)	C12^i^—C11—C7	121.54 (17)
C3—C4—N1	118.7 (3)	C12—C11—C7	121.54 (17)
C5—C4—N1	120.6 (4)	C13—C12—C11	121.9 (3)
C4—C5—C6	119.7 (4)	C13—C12—H12	119.0
C4—C5—H5	120.2	C11—C12—H12	119.0
C6—C5—H5	120.2	C14—C13—C12	119.0 (3)
C5—C6—C1	121.6 (4)	C14—C13—H13	120.5
C5—C6—H6	119.2	C12—C13—H13	120.5
C1—C6—H6	119.2	C13—C14—C13^i^	121.2 (3)
C1—C7—C11	109.6 (3)	C13—C14—N2	119.38 (18)
C1—C7—C8	109.2 (2)	C13^i^—C14—N2	119.38 (18)
C11—C7—C8	110.63 (19)	C4—N1—H1A	108.8
C1—C7—C8^i^	109.2 (2)	C4—N1—H1B	109.5
C11—C7—C8^i^	110.63 (19)	H1A—N1—H1B	108.6
C8—C7—C8^i^	107.5 (3)	C4—N1—H1C	109.3
C9—C8—C7	112.4 (3)	H1A—N1—H1C	111.0
C9—C8—H8A	109.1	H1B—N1—H1C	109.6
C7—C8—H8A	109.1	C14—N2—H2A	109.5
C9—C8—H8B	109.1	C14—N2—H2B	109.5
C7—C8—H8B	109.1	H2A—N2—H2B	109.5
H8A—C8—H8B	107.8	C14—N2—H2C	109.5
C8—C9—C10	111.2 (3)	H2A—N2—H2C	109.5
C8—C9—H9A	109.4	H2B—N2—H2C	109.5
C10—C9—H9A	109.4	H1D—O1—H1E	90.4
C8—C9—H9B	109.4		
			
C6—C1—C2—C3	0.0	C1—C7—C8—C9	−175.1 (2)
C7—C1—C2—C3	180.0	C11—C7—C8—C9	64.2 (3)
C1—C2—C3—C4	0.0	C8^i^—C7—C8—C9	−56.7 (4)
C2—C3—C4—C5	0.0	C7—C8—C9—C10	56.5 (3)
C2—C3—C4—N1	180.0	C8—C9—C10—C9^i^	−53.9 (5)
C3—C4—C5—C6	0.0	C1—C7—C11—C12^i^	88.2 (3)
N1—C4—C5—C6	180.0	C8—C7—C11—C12^i^	−151.3 (3)
C4—C5—C6—C1	0.0	C8^i^—C7—C11—C12^i^	−32.3 (4)
C2—C1—C6—C5	0.0	C1—C7—C11—C12	−88.2 (3)
C7—C1—C6—C5	180.0	C8—C7—C11—C12	32.3 (4)
C2—C1—C7—C11	0.0	C8^i^—C7—C11—C12	151.3 (3)
C6—C1—C7—C11	180.0	C12^i^—C11—C12—C13	−1.0 (5)
C2—C1—C7—C8	−121.3 (2)	C7—C11—C12—C13	175.6 (3)
C6—C1—C7—C8	58.7 (2)	C11—C12—C13—C14	0.6 (5)
C2—C1—C7—C8^i^	121.3 (2)	C12—C13—C14—C13^i^	−0.2 (6)
C6—C1—C7—C8^i^	−58.7 (2)	C12—C13—C14—N2	−179.2 (3)

Symmetry codes: (i) *x*, −*y*+1/2, *z*.

### Hydrogen-bond geometry (Å, °)

**Table:**

*D*—H···*A*	*D*—H	H···*A*	*D*···*A*	*D*—H···*A*
N2—H2C···Cl1^ii^	0.89	2.70	3.253 (3)	121.
N2—H2C···Cl1^iii^	0.89	2.53	3.252 (3)	139.
N2—H2B···Cl1^iv^	0.89	2.41	3.253 (3)	159.
N2—H2A···Cl1^v^	0.89	2.46	3.252 (3)	148.
O1—H1E···Cl1^vi^	0.85	2.42	3.183 (3)	150.
O1—H1D···Cl1^vii^	0.82	2.38	3.183 (3)	167.
N1—H1C···Cl1^viii^	0.90	2.22	3.101 (3)	165.
N1—H1B···O1^ix^	0.90	1.78	2.678 (6)	172.
N1—H1A···Cl1^x^	0.90	2.23	3.101 (3)	163.

Symmetry codes: (ii) *x*+1, −*y*+3/2, *z*; (iii) −*x*+2, *y*−1/2, −*z*+1; (iv) *x*+1, *y*−1, *z*; (v) −*x*+2, −*y*+1, −*z*+1; (vi) −*x*+1, −*y*+1, −*z*+1; (vii) −*x*+1, *y*−1/2, −*z*+1; (viii) *x*+1, *y*−1, *z*−1; (ix) *x*, *y*, *z*−1; (x) *x*+1, −*y*+3/2, *z*−1.
